# Negative Pressure Wound Therapy with Instillation in a Chronic Non-Healing Right Hip Trochanteric Pressure Ulcer

**DOI:** 10.7759/cureus.877

**Published:** 2016-11-14

**Authors:** Kevin W Broder, Brian Nguyen, Richard M Bodor

**Affiliations:** 1 Section of Plastic Surgery VA Healthcare - San Diego, Division of Plastic Surgery University of California San Diego; 2 School of Medicine, University of California, San DIego

**Keywords:** technology, wound care, npwt, npwti-d, npwt with instillation, pressure sores, pressure ulcers, flap reconstruction, plastic surgery, negative pressure wound therapy with instillation

## Abstract

Complex pressure ulcer wound sites often present with a wide scope of barriers to healing ranging from high colonization of multi-drug-resistant pathogens to tortuous internal anatomy which make the wound recalcitrant to traditional wound care including standard negative pressure wound therapy (NPWT). Negative pressure wound therapy with instillation (NPWTi-d) provides an opportunity to manage and heal wounds with indications not met by standard NPWT such as cavitating wounds with complex undermining and tunneling. In this clinical case report, a patient who presented with a chronic, non-healing Stage IV pressure ulcer underwent a tensor fascia lata flap reconstruction that was complicated by a partial flap-tip nonadherence with associated partial dehiscence of the flap incision that proved unresolvable until application of adjunctive NPWTi-d which allowed the wound to experience a robust rate of granulation, contraction, and closure.

## Introduction

Pressure ulcers are a highly prevalent source of morbidity with an equally high incidence of up to 38.0% amongst different categories of healthcare institutions [1]. Therefore, the management and therapeutic approach toward these often hospital- or facility-acquired problems remain critical aspects of long-term care [2]. Often, complexities exist structurally within these wounds including undermining, tunneling, and sinus tract formation along with exudate and necrotic tissue [3]. These serve as barriers to healing as they may host resident and occult sources of foreign bodies as well as unreachable nonviable material, both of which may promote ischemia, inflammatory responses, and an increased susceptibility to pathogenic invasion [4-8].

Negative pressure wound therapy (NPWT) has proven effective in addressing the barriers to pressure ulcer healing including increasing blood flow to previously ischemic wound areas by generating subatmospheric pressure which vacuums in circulation [4]. Pro-inflammatory cytokines and enzymes are furthermore decreased [5-6], while favorable healing factors such as the infiltration of VEGF and chemotaxis of fibroblasts increase angiogenesis [7-8]. Due to the mechanical washout of the wound bed, the pathogenic load is decreased thus indirectly lowering the toxic burden on the pressure wound [4].

Negative pressure wound therapy with instillation (NPWTi-d) presents as an innovative treatment modality to introduce irrigating fluids into previously difficult-to-access undermining and tunneling in cavitating wounds. Compared to standard NPWT in an agar-based wound model, NPWTi-d displayed statistically significant doubling of wound-bed coverage [9]. NPWTi-d advances bacterial clearance as well as induces up to 43% more granulation tissue than traditional NPWT [10]. In this case report, we present a patient with a chronic pressure ulcer with a complex internal structure that did not respond to traditional negative pressure wound therapy, but showed a robust response and resolution with the added NPWTi-d application.

## Case presentation

A 57-year-old, paraplegic woman presented to the hospital with a right hip trochanteric Stage IV pressure ulcer down to the level of exposed bone. The skin defect was located on the posterior thigh, and the wound tracked to the trochanteric bursa. Operative irrigation and debridement of the wound were performed with intraoperative wound and bone cultures demonstrating no growth. The pressure ulcer was nonresponsive to traditional moist wound-care measures which included standard NPWT with silver granufoam, nutrition optimization, and pressure-relief strategies including lying on an air-fluidized bed. A tensor fascia lata flap reconstruction was then performed, but the post-operative course was complicated by a partial flap tip nonadherence with associated partial dehiscence of the flap incision. The patient refused recommendations for further flap advancement; therefore, standard NPWT was continued. However, over the next two months, wound healing failed to progress. Without response to standard NPWT, NPWTi-d was initiated with the V.A.C. VeraFlo Therapy (Acelity, San Antonio, TX). ​Wound volume rapidly decreased after NPTWTi-d was started with complete wound closure in seven weeks.

Veraflo settings for this patient were: Irrigant: normal saline Target Pressure: 125 mmHg Intensity: low Soak Time: 15 minutes Therapy Time: 4 hours

## Discussion

**Figure 1:**
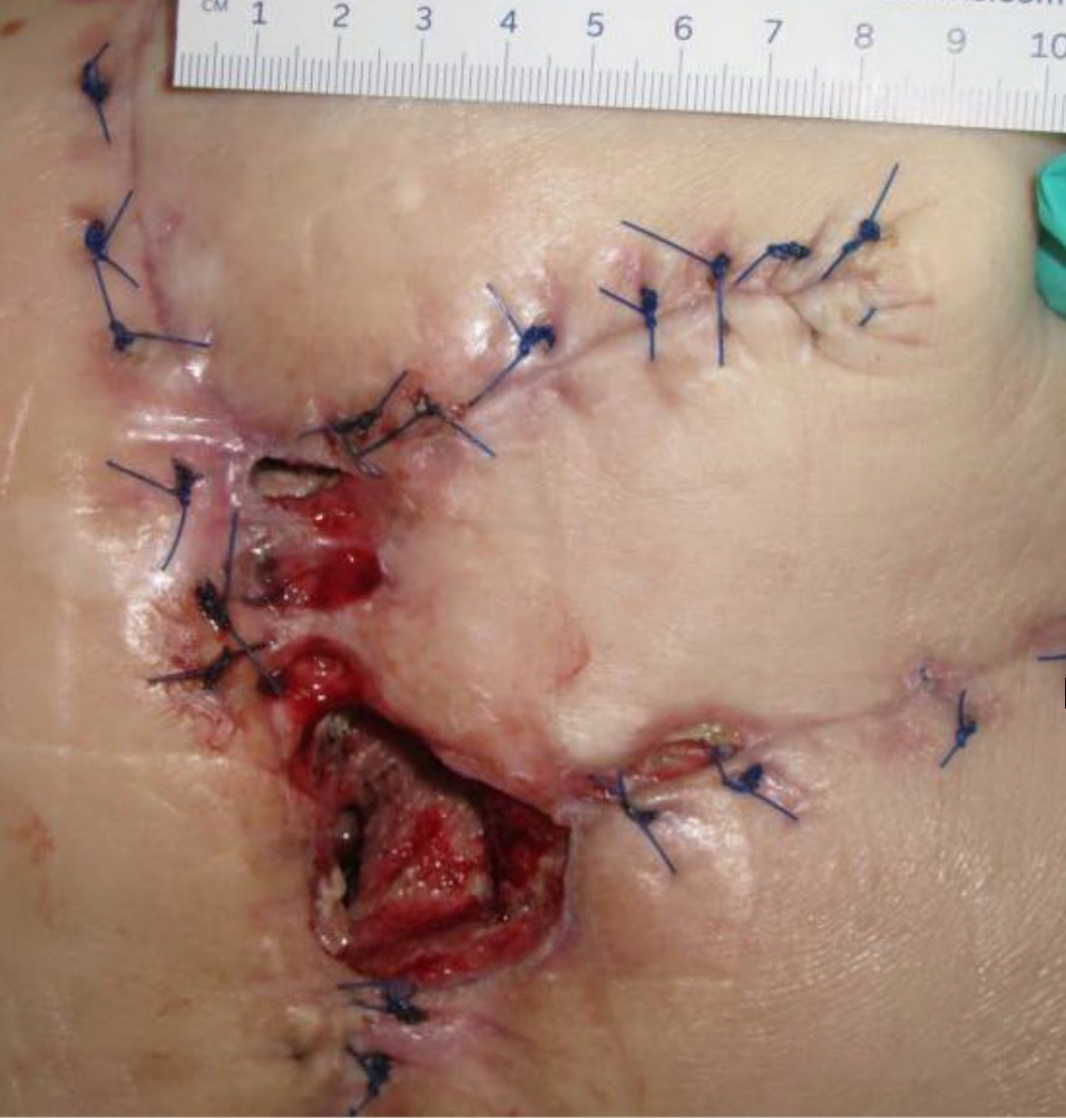
Day 1 3 cm x 1.5 cm x 7.5 cm (Length x Width x Depth) 33.75 cm^3^ (Volume)

**Figure 2:**
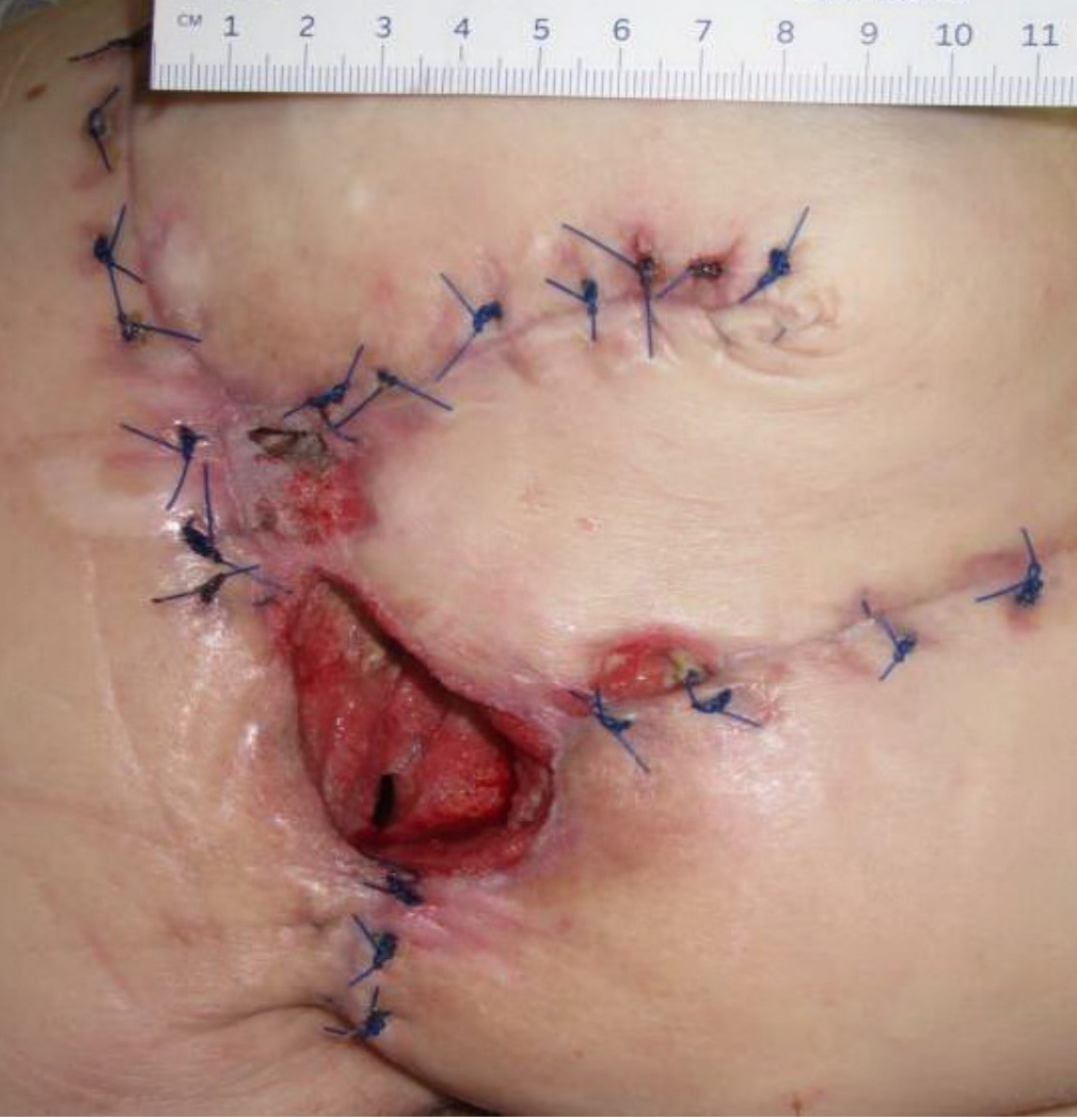
Day 8 4.0 cm x 3.8 cm x 4.7 cm (Length x Width x Depth) 71.44 cm^3^ (Volume)

**Figure 3:**
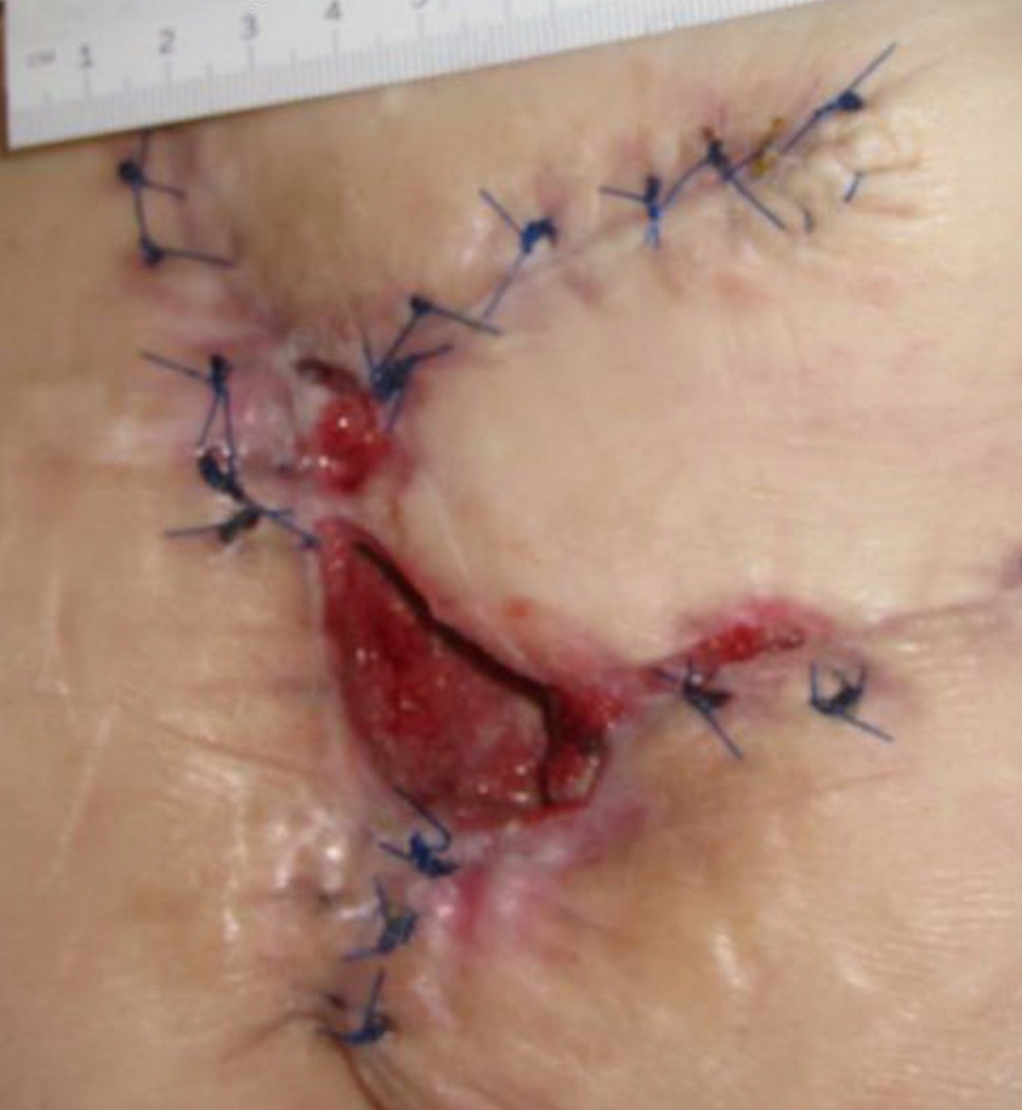
Day 15 4.0 cm x 3.5 cm x 6.5 cm (Length x Width x Depth)​​ 91 cm^3^ (Volume)

**Figure 4:**
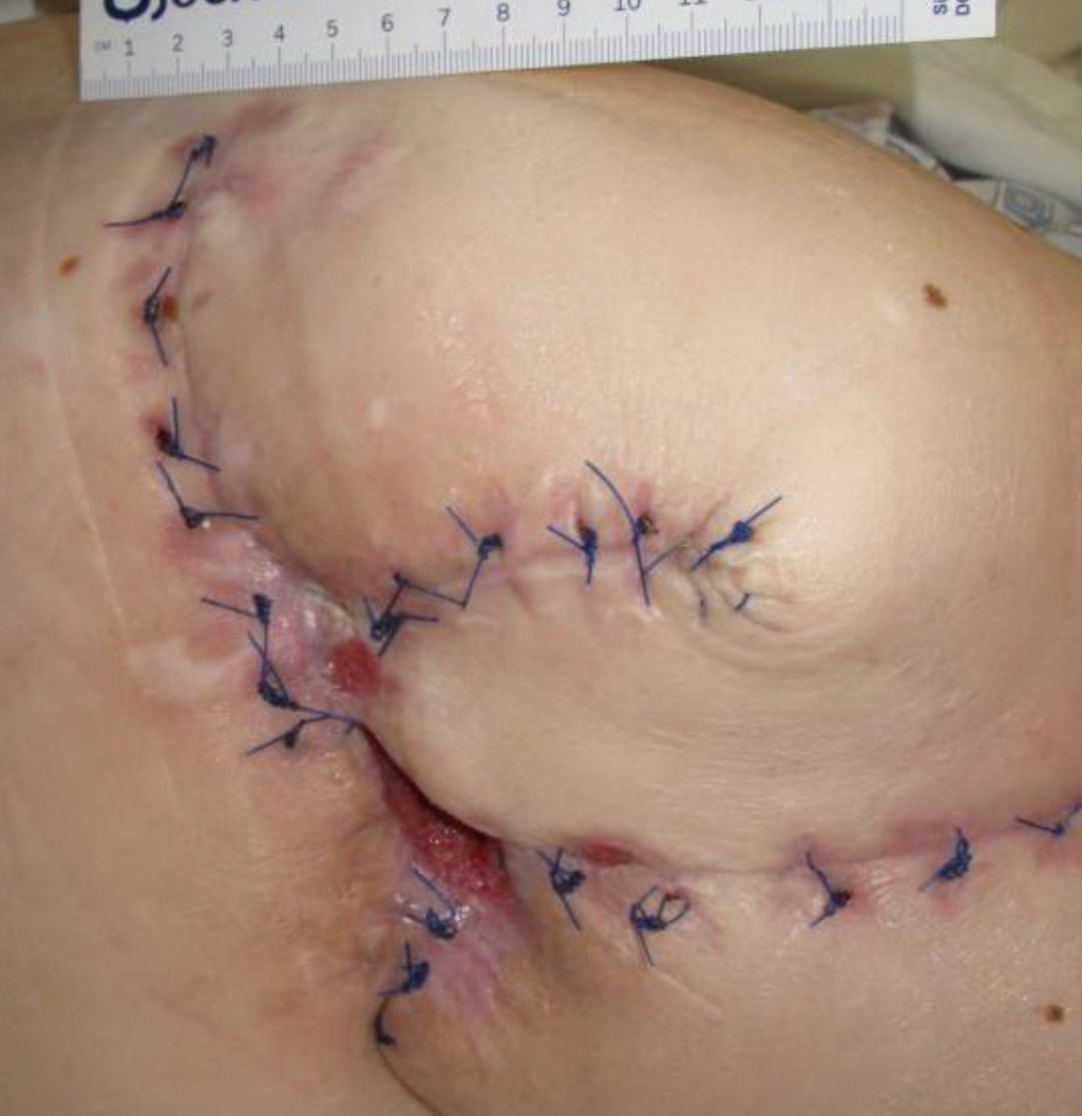
Day 29 3.8 cm x 3.2 cm x 5.0 cm (Length x Width x Depth) 60.80 cm^3^ (Volume)​

**Figure 5:**
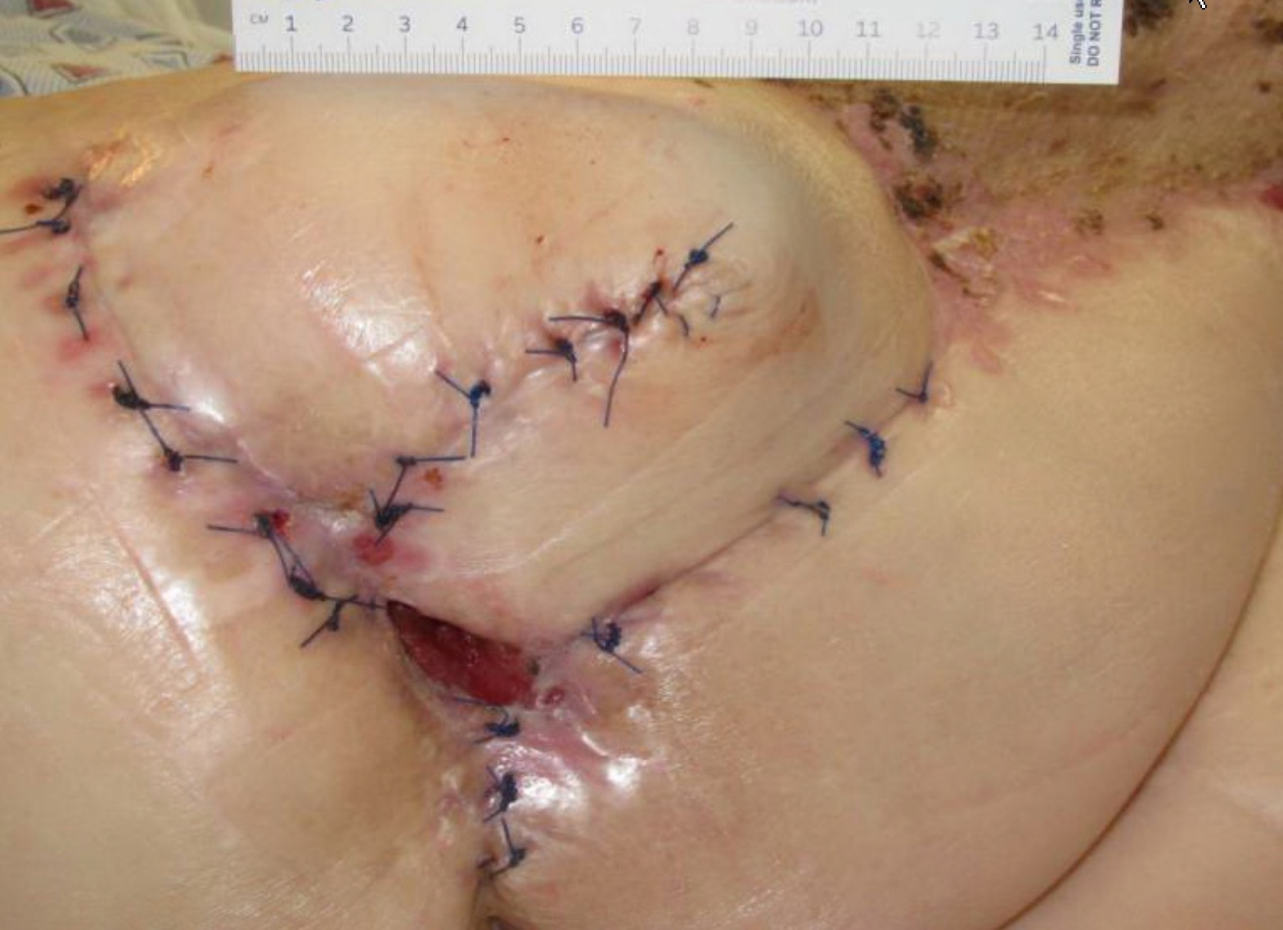
Day 43 2.2 cm x 3.0 cm x 2.5 cm (Length x Width x Depth) 16.50 cm^3^ (Volume)​

**Figure 6:**
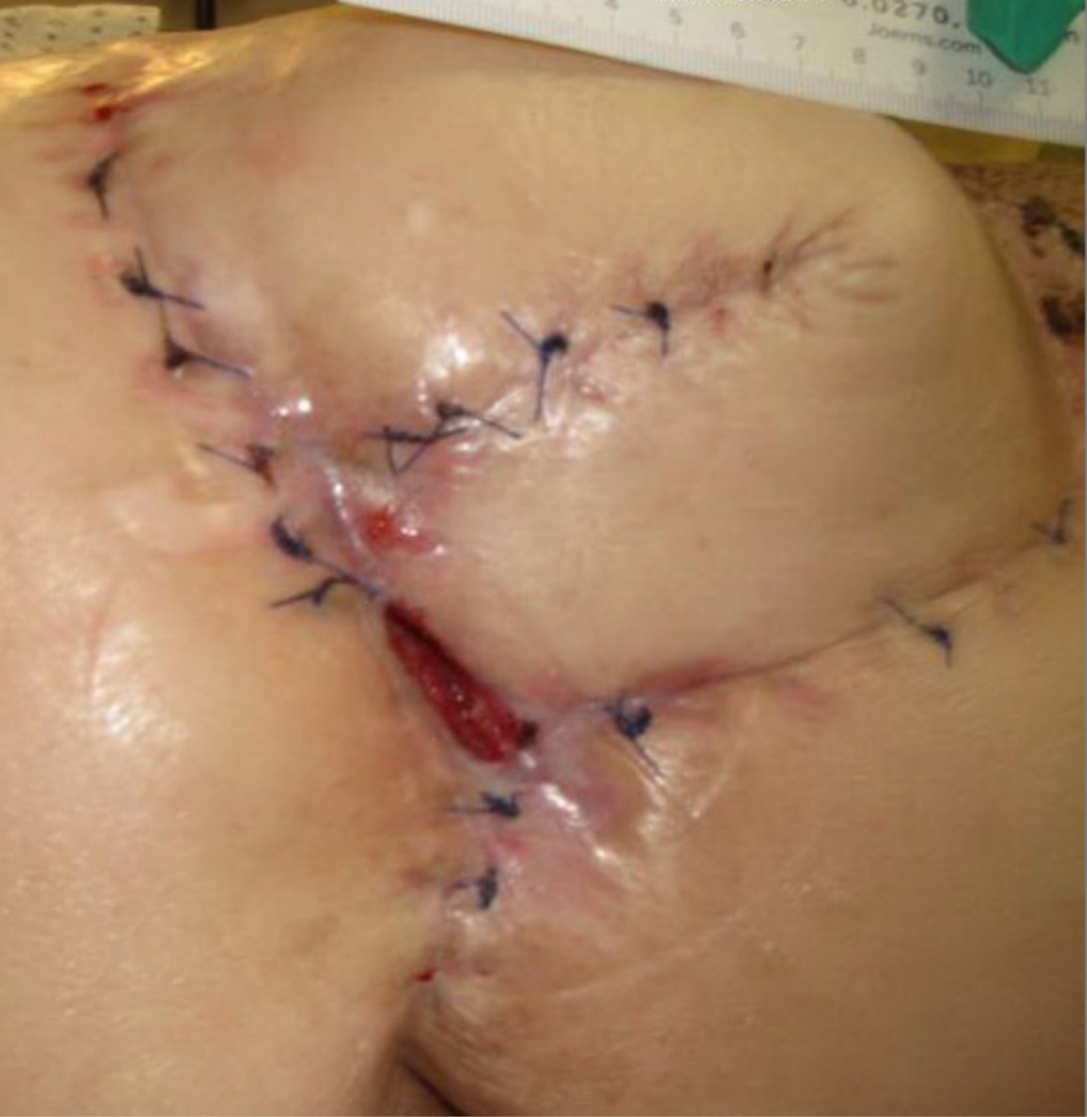
Day 50 0.6 cm x 2.7 cm x 0.7 cm (Length x Width x Depth) 1.13 cm^3^ (Volume)​

**Figure 7:**
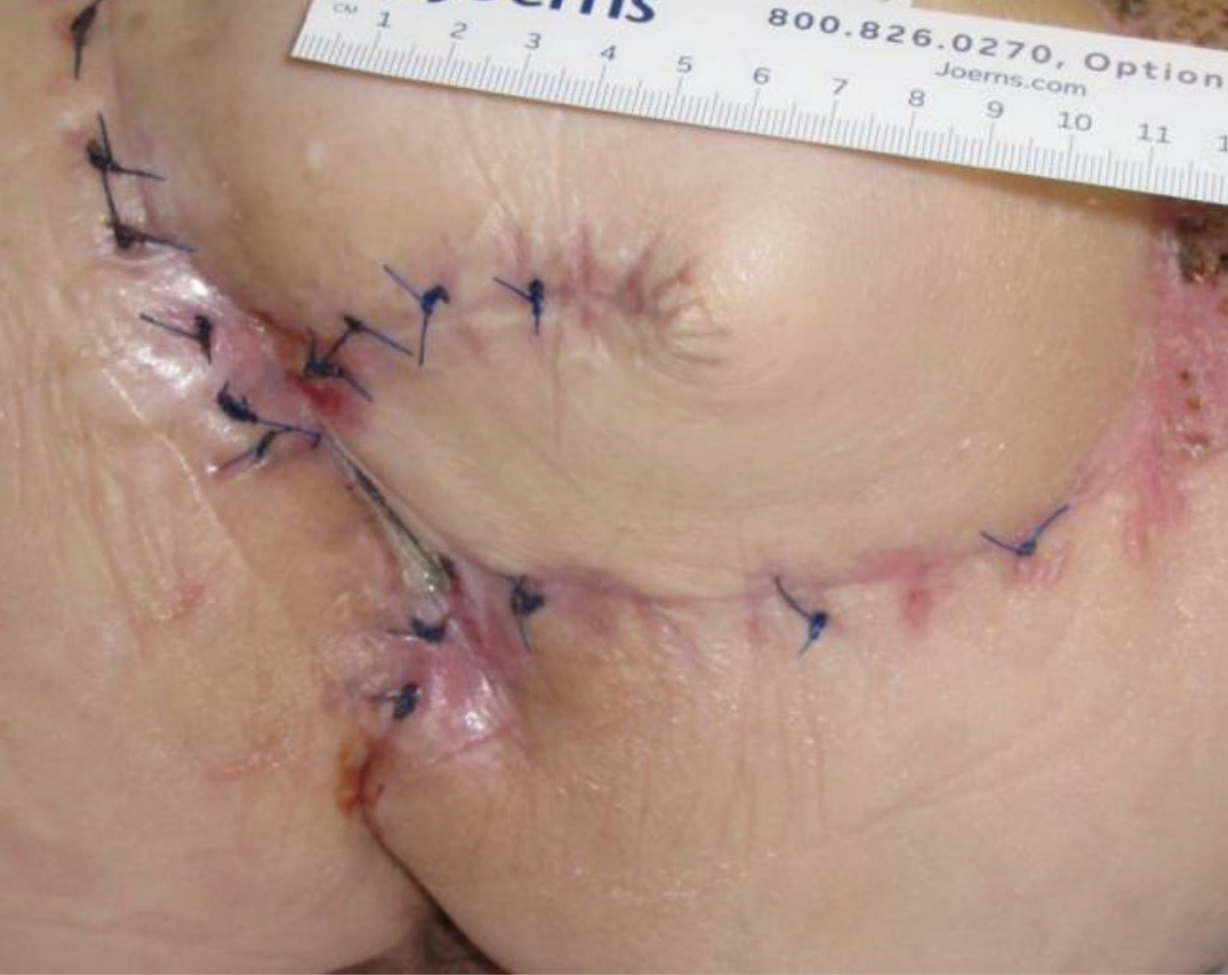
Day 57 0.5 cm x 0.2 cm x 0.0 cm (Length x Width x Depth) 0.00 cm^3^ (Volume)​

**Figure 8:**
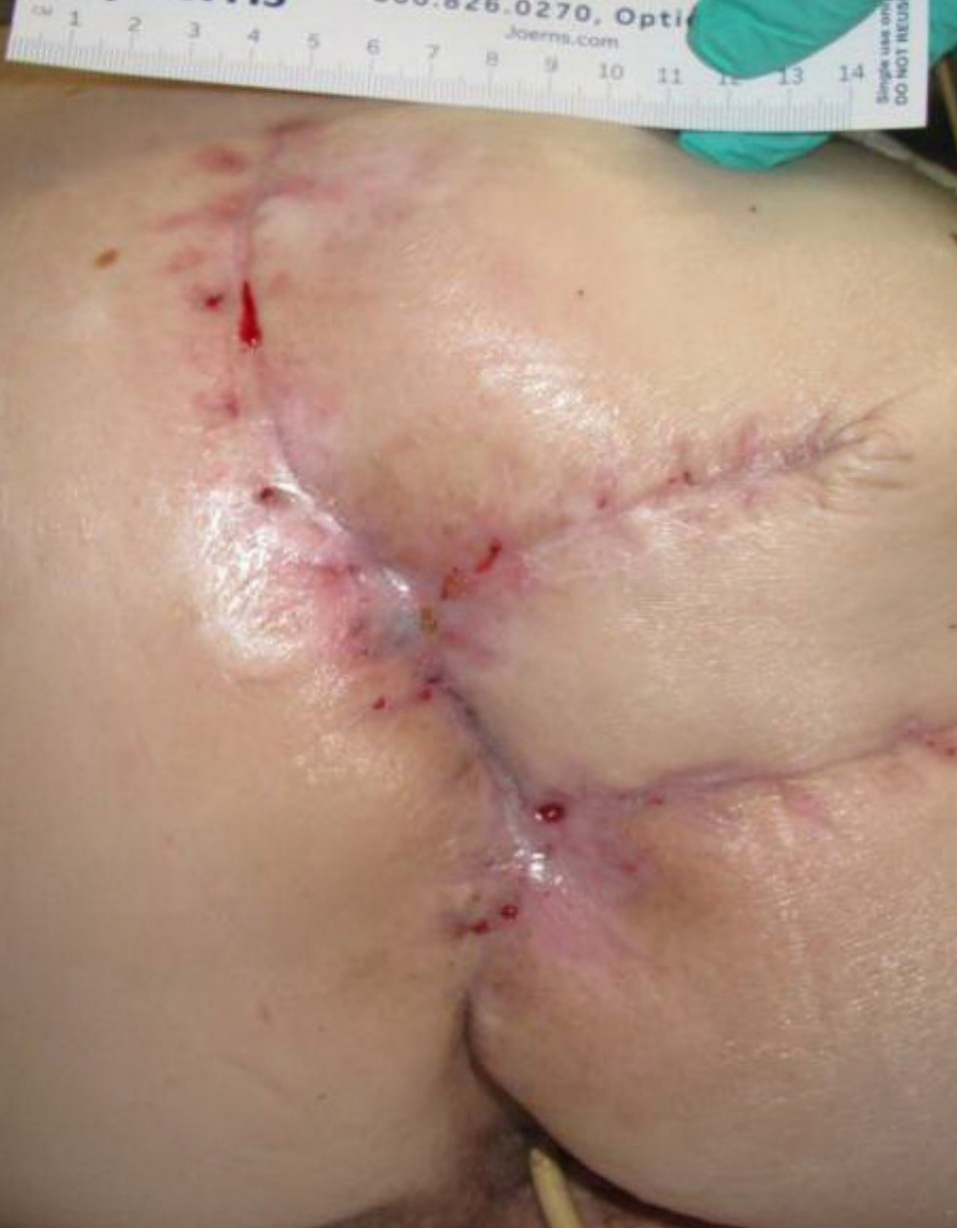
Day 64 Healed

**Figure 9:**
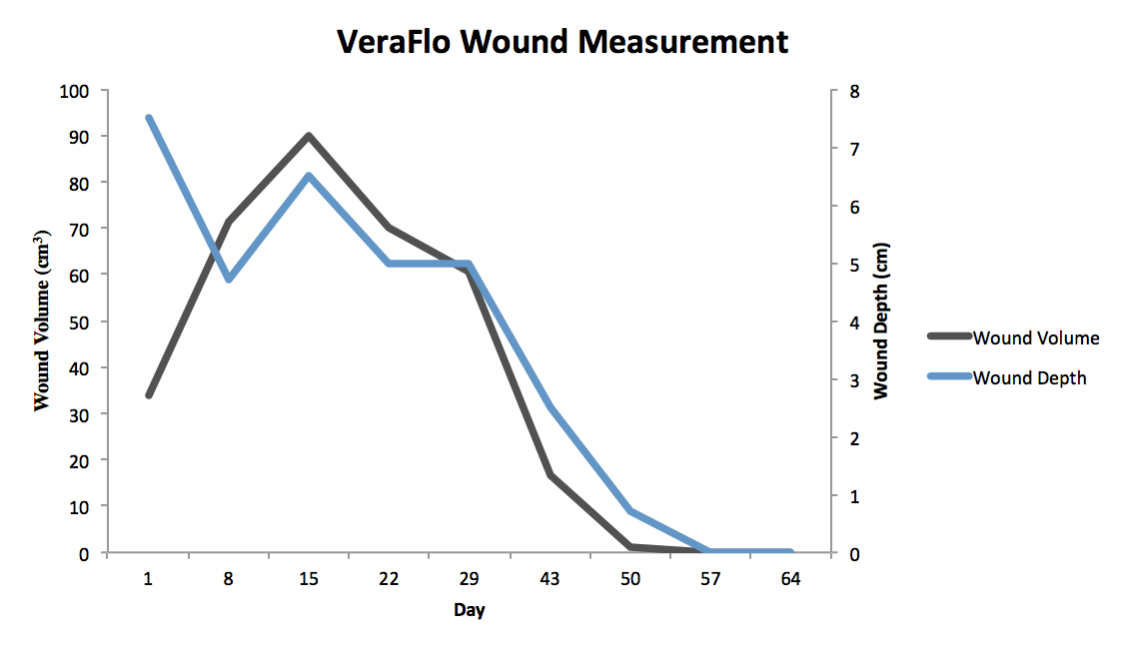
VeraFlo Wound Measurement Volume and depth measurements across treatment timeline.

The patient presented with a Stage IV trochanteric pressure ulcer with exposed bone and associated highly complex labyrinthine internal structure of undermining and deep tunneling. With the inherent complex structure of the wound combined with her refusal of recommended continued operative treatment, NPWTi-d represented an appropriate alternative option to deliver adjunctive therapy to the wound bed. Once NPWTi-d was applied, there was a progressive decrease in wound dimension (Figures 1-9). Some variability in obtaining consistent wound measurements may be related to patient positioning and the complex three-dimensional structure of the wound. This likely explains the initial increase in wound volume measured.

The patient agreed to participate and was explained the nature and objectives of this study, and informed consent was formally obtained. No reference to the patient's identity was made at any stage during data analysis or in the report.

## Conclusions

This case report was presented due to its representative nature of common, chronic, non-healing pressure ulcers, which was resolved with an unconventional and novel application of NPWTi-d technology. The incidence and prevalence of pressure ulcers remain high amongst many institutions, and yet, despite a variety of treatment modalities, robust closure of the complicated wound bed remains difficult. NPWTi-d presents several advantages over traditional NPWT including enhanced bacterial clearance, increased granulation formation, and augmented therapeutic penetration into previously unaccessed inner-wound-bed anatomy. NPWTi-d thus may present a viable solution for chronic, non-healing pressure ulcers with many indications not met by traditional negative wound therapy, ranging from complex internal structures of cavitating wounds to refusal of operative treatment.
